# Ceratohyoidectomy for Management of Unilateral and Bilateral Temporohyoid Osteoarthropathy: Long-Term Outcome in 33 Equids

**DOI:** 10.3390/ani16152318

**Published:** 2026-07-28

**Authors:** Heather S. McCrary, Holly L. Stewart, Joseph G. Davis, Jose M. Garcia-Lopez, Kirstin A. Bubeck

**Affiliations:** 1 Department of Clinical Sciences, Cummings School of Veterinary Medicine at Tufts University, North Grafton, MA 01536, USA; heather.mccrary@tufts.edu (H.S.M.); joseph.davis@tufts.edu (J.G.D.); 2 Department of Clinical Studies-New Bolton Center, University of Pennsylvania, Kennett Square, PA 19348, USA; hstew@upenn.edu (H.L.S.); jmgarcia@vet.upenn.edu (J.M.G.-L.)

**Keywords:** horse, temporohyoid osteoarthropathy, THO, ceratohyoidectomy, neurologic, computed tomography, endoscopy, facial nerve paralysis, ataxia

## Abstract

Temporohyoid osteoarthropathy is a disease affecting the temporohyoid joint in equids caused by local inflammation and/or degeneration, leading to neurological deficits ranging from mild signs that affect performance to significant, acute neurological deficits such as facial nerve paralysis or vestibular signs. Multiple surgical treatment options are available, and while treatment has historically been performed unilaterally on the side of the clinical signs, advances in diagnostic imaging have highlighted the occurrence of bilateral disease of the temporohyoid joints, warranting evaluation of bilateral surgical treatment. The objective of this study is to report the findings, surgical complications, and outcomes of both unilateral and bilateral ceratohyoidectomy in 33 equids diagnosed with THO. This study found that there was no significant difference in outcome based on which procedure was performed, but that increased age and longer duration of clinical signs were associated with decreased likelihood of horses returning to work or showing clinical improvement. Overall, this study reports 84% survival to long term; 86% cases showed improvement, with 38% showing complete resolution of signs, 62% returned to the previous level of exercise, and 77% returned to work at the same or lower level.

## Simple Summary

Temporohyoid osteoarthropathy is a disease affecting the temporohyoid joint in equids caused by local inflammation and/or degeneration, leading to neurological deficits. Multiple treatments have been described, including medical management and various surgical approaches. Historically, a unilateral approach is often recommended; however, bilateral treatment has been reported. The objective of this retrospective study is to report the outcomes of 33 equids following either a unilateral or a bilateral ceratohyoidectomy. This study shows that although the outcomes of the two approaches are not different, both are reasonable options when considering surgical intervention for this disease.

## 1. Introduction

Temporohyoid osteoarthropathy (THO) is a disease affecting the temporohyoid joint (THJ) in horses caused by local infection and/or inflammation of the inner ear or guttural pouch, although the exact etiology is unknown [1,2,3,4,5,6,7]. Degenerative changes to the THJ are thought to contribute to joint enlargement, periarticular inflammation, reduced stylohyoid movement, and ankylosis [5,8,9]. Fractures of the temporal portion of the skull or stylohyoid bone can occur, resulting in inflammation affecting the nearby nerves [9,10,11,12,13]. Clinical signs include pain on palpation of the basihyoid region or base of the skull, ear drooping, muzzle deviation, exposure keratitis, ataxia, head tilt, circling, nystagmus, and dysphagia [1,2,6,12,13,14,15,16,17,18,19].

Treatment options include medical management, which often fails; therefore, surgery is considered the treatment of choice [3,12,20,21]. Current surgical treatment options include basi-ceratohyoid joint disarticulation and ceratohyoidectomy (CHO). This procedure involves removing the ceratohyoid bone completely or partially, either under general anesthesia or standing sedation. The proposed result is a reduction in motion transferred through the hyoid apparatus from the tongue, mouth, and neck, resulting in less force applied to the THJ. Changes to the THJ are commonly seen bilaterally on computed tomography [5], and horses undergoing unilateral CHO can present later with THO on the contralateral side [21,22,23,24]. If bilateral disease is present, the current recommendation is to perform the procedure on the side with the main clinical deficits, delaying treatment of the contralateral side until clinical signs appear [24].

The objective of this study is to report the short- and long-term outcomes, as well as intra- and postoperative complications, of unilateral and bilateral CHO performed in horses with unilateral or bilateral THO. We hypothesized that horses undergoing bilateral CHO would have a similar long-term outcome as horses undergoing unilateral CHO. A secondary hypothesis was that horses with more severe endoscopy and CT grades would have a worse outcome.

## 2. Materials and Methods

### 2.1. Case Selection

**Figure 1:**
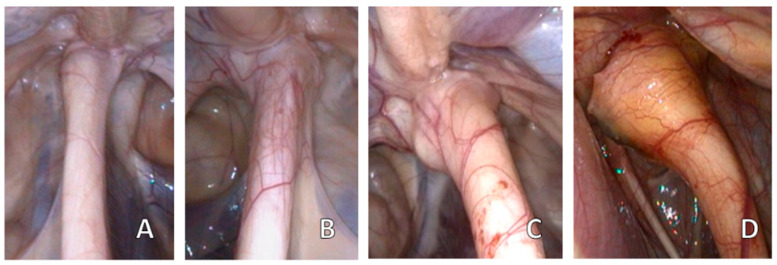
Examples of each grade based on endoscopy: (**A**) grade 0; (**B**) grade 1; (**C**) grade 2; (**D**) grade 3.

**Figure 2:**
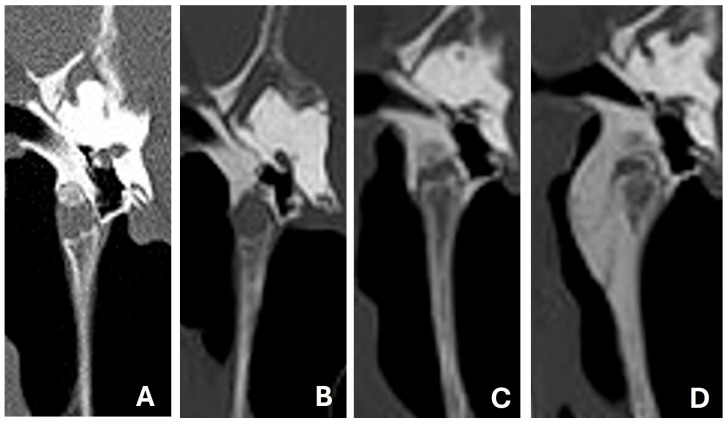
Examples of CT images for each grade: (**A**) grade 0; (**B**) grade 1; (**C**) grade 2; (**D**) grade 3.

Equids diagnosed with THO and undergoing CHO at the Hospital for Large Animals, Cummings School of Veterinary Medicine at Tufts University, between January 2009 and December 2025 were included in the analysis. Case records were reviewed, and the age, breed, sex, clinical history, presenting complaint, diagnostic imaging performed at intake, affected side, surgical treatment (bilateral vs. unilateral CHO), intra- and postoperative complications, and duration of hospitalization were recorded. Diagnostic imaging included radiographs, endoscopy, and computed tomography (CT), in addition to the clinical assessment and neurological exam, to reach a diagnosis of THO. Endoscopy images available (21 cases) were evaluated and graded by three blinded board-certified large animal surgeons. Endoscopy images were graded based on a previously reported grading scale [21], where Grade 0: normal, Grade 1: mild enlargement of the stylohyoid bone and THJ, Grade 2: moderate enlargement of the stylohyoid bone and THJ, and Grade 3: severe enlargement of the stylohyoid bone and THJ (Figure 1). All CT images were reviewed by two blinded board-certified radiologists. The CT images were graded based on the severity of changes in the THJ and stylohyoid bone using a previously reported grading scale [11,25] where Grade 0: no proliferation, Grade 1: proliferation not bridging the joint, Grade 2: proliferation bridging the joint, but extending over less than 1 cm, and Grade 3: proliferation over more than 1 cm (Figure 2). Follow-up for each case was obtained from medical records, information from the referring veterinarian, and via a questionnaire sent to owners. Short-term outcome was defined as less than one year, and long-term outcome was defined as greater than one year post-surgery.

### 2.2. Surgical Procedure

All horses were administered broad-spectrum antimicrobials (ceftiofur sodium 2.2 mg/kg or potassium penicillin 20,000 IU/kg IV and gentamicin 6.6 mg/kg IV) and a non-steroidal anti-inflammatory (NSAID) (phenylbutazone 4.4 mg/kg IV or flunixin meglumine 1.1 mg/kg IV) prior to surgery followed by phenylbutazone 2.2 mg/kg IV q12h or flunixin meglumine 1.1 mg/kg IV q12h for 24 h postoperatively. The choice of antimicrobial was at the discretion of the attending clinician. Intraoperatively, each equid received 0.04–0.05 mg/kg dose of intravenous dexamethasone. 

Horses were sedated with xylazine 1.1 mg/kg IV titrated to effect or detomidine 0.01–0.03 mg/kg IV and butorphanol 0.01–0.02 mg/kg IV prior to induction with 2.2 mg/kg ketamine IV and 0.05–0.08 mg/kg midazolam IV. After orotracheal intubation, they were placed in dorsal recumbency and maintained on isoflurane. The surgical approach used for CHO has been previously described [20]. An approximately 10 cm longitudinal incision medial to the linguofacial vein was made on the affected side, followed by careful dissection to the ceratohyoid–basihyoid articulation. This articulation was freed using careful dissection with a periosteal elevator and blunt dissection using Metzenbaum scissors. Once freed, the basi-ceratohyoid joint was transected. Blunt dissection with a periosteal elevator was followed dorsally along the ceratohyoid bone until reaching the ceratohyoid–stylohyoid articulation, which was carefully disarticulated using the periosteal elevator. This was followed by the removal of the ceratohyoid bone. Care was taken during the procedure to avoid the hypoglossal nerves, lingual branches of the mandibular and glossopharyngeal nerves, and lingual vessels. If the procedure was performed bilaterally, the same steps were repeated for the contralateral side. Variation in approach for bilateral CHO was at the discretion of the performing surgeon, with the option of making one midline incision or two incisions on each side. The surgical site was then thoroughly lavaged with sterile saline. The incision was closed in three layers, and a bandage was applied using sterile gauze and iodine-impregnated surgical drape (3M™ Ioban™ 2 Antimicrobial Incise Drape, Solventum Corporation, Eagan, MN, USA). All patients were recovered intubated, and extubation was delayed until they were standing.

All horses were discharged based on clinical assessment and were prescribed an oral antimicrobial (trimethoprim sulfamethoxazole 30 mg/kg PO q12h for 3 days unless other conditions required further administration), and an oral NSAID (phenylbutazone 2.2 mg/kg PO q12h for 3 days followed by 2.2 mg/kg PO q24h for 4 days). Exercise instructions included stall rest for 4 weeks, with gradually increasing hand walks and turnout. For horses undergoing bilateral CHO, prolonged exercise restriction was advised for 3–4 months to allow fibrous connective tissue to develop at the surgical site and reduce the likelihood of epiglottic retroversion. Horses could be hand-walked and have small paddock turnout but not undergo any forced exercise. It was recommended that each horse be rechecked by a veterinarian prior to returning to full exercise.

### 2.3. Statistical Analysis

All data was organized in Microsoft Excel for analysis. Categorical variables were reported as frequency count (percentage of total), and continuous variables were assessed for normality using the Shapiro–Wilk test. All descriptive continuous data were non-parametric and reported as median and range. Univariable logistic regression was used to assess the association between independent explanatory variables and outcomes. Outcomes of interest were clinical improvement and return to work. In cases of complete separation between variables and outcomes, Firth penalized logistic regression was used. Multicollinearity was assessed using variance inflation factors and R^2^ for each independent variable. Independent variables with evidence of association (defined as *p* ≤ 0.2) in univariable logistic regression analysis were selected for multivariable logistic regression analysis. The goodness-of-fit of the model was assessed with the Akaike information criterion (AIC). The final model was constructed using stepwise backward selection to remove non-significant independent variables. Variables were considered significantly associated with the outcome of interest when *p* < 0.05. Datasets for unilateral and bilateral CHO cases were first assessed separately and then combined due to low case numbers. Uni- and multivariate regression models are described as odds ratios (ORs) and 95% confidence intervals (CIs). Interobserver agreement was assessed for CT and endoscopy scores. All data were analyzed using R software (version 4.2.2, R Foundation of Statistical Computing 2022) in RStudio (version 2022.12.0+353) using the base package for data comparison and regression modeling.

## 3. Results

### 3.1. Signalment and History

A total of 33 patients met the inclusion criteria. Median age was 15 years old (range 5–28 years); median body weight was 517 kg (range 119–642 kg). Horses were used for jumping (*n* = 8, 24%), pleasure riding (*n* = 8, 24%), dressage (*n* = 4, 12%), and barrel racing (*n* = 1, 3%). The use of eight horses and the donkey (27%) was unspecified, and three horses (9%) had been previously retired. Case signalment can be found in Table 1.

### 3.2. Clinical Signs

The most common clinical sign at presentation was ataxia (*n* = 13, 39%), followed by facial nerve paralysis (*n* = 12, 36%) and head tilt (*n* = 11, 33%). Other less common clinical signs included nystagmus (*n* = 6, 18%), dysphagia (*n* = 4, 12%), difficulty chewing or holding a bit (*n* = 4, 12%), loss of tongue tone (*n* = 3, 9%), and poor performance (*n* = 3, 9%).

Thirteen (39%) horses presented with clinical signs on the right, six (18%) with signs on the left, three (9%) with bilateral signs, and eleven (33%) with clinical signs of unknown laterality or non-lateralizing signs. Bilateral signs included: head tilt to the right and left masseter atrophy; right head tilt and right facial nerve deficits with gait deficits due to left vestibular disease. A third horse showed bilateral gait deficits (left worse than right), with left ear droop and rightward muzzle deviation, and bilateral masseter atrophy.

The duration of clinical signs before presentation was available for 30 horses and ranged from 1 to 365 days (mean 82.5 ± 130.14 days; median 28 days).

### 3.3. Diagnostic Findings

#### 3.3.1. Endoscopy

Twenty-seven (81%) horses were examined via upper airway endoscopy of the guttural pouches, and information from medical records was included for analysis. Endoscopic changes included mild to severe thickening of the stylohyoid bone and the THJ, with changes on the right in 8 (29%) cases, on the left in 6 (21%) cases, and bilaterally in 11 (40%) cases. In one (3%) case, the guttural pouches could not be entered due to soft tissue swelling, and in one case, there were no endoscopically visible changes. Three cases (9%) were not evaluated endoscopically at presentation. In these three cases, horses had been endoscopically evaluated by the referring veterinarian and were referred for CT.

#### 3.3.2. Endoscopic Image Grades

Endoscopic images of 21 horses were available for retrospective grading.

Median endoscopic grade for the left was 2 (range 0–3), and for the right it was 1.5 (range 0–3). Interobserver agreement for endoscopy scores ranged from fair to moderate (left: kappa = 0.517; right: kappa = 0.422).

#### 3.3.3. CT Examination

Twenty-nine (88%) horses underwent CT examination prior to surgery, and all images were available for review. CT changes included irregular periarticular bone formation at the THJs, with some having fracture of either the stylohyoid bone (*n* = 6, 21%) or the petrous part of the temporal bone (*n* = 1, 3%). Reviewing the medical records, right-sided changes were noted in seven (24%) cases, left-sided changes were noted in four (14%) cases, and bilateral changes were noted in 18 (62%) cases. Other findings included soft tissue swelling adjacent to the joint and findings consistent with otitis media (soft tissue attenuation in the tympanic bulla, enlargement of the tympanic bulla, and thickening of the ear canal) in six (21%).

#### 3.3.4. CT Grades

When CT images available for 29 equids were graded by two radiologists retrospectively, 28 horses (97%) were found to have bilateral changes, with only one horse graded as grade 0 on the left side and grade 3 on the right side (case 8). Median CT score for the left was 2.5 (range 0–3), and for the right it was 2.75 (range 0.5–3). Interobserver agreement for CT scores ranged from moderate to good (left: kappa = 0.657; right: kappa = 0.519). Four (12%) cases did not undergo CT because of previously diagnosed THO via endoscopy.

### 3.4. Surgical Outcomes

Seventeen (52%) horses underwent a unilateral CHO. Thirteen (41%) were treated on the right and four (13%) were treated on the left. Sixteen (48%) horses were treated bilaterally. Case 26 had bilateral CHO without complications, but the owner elected euthanasia on the table after assessment of a mass within the left guttural pouch. Case 29 was treated with a unilateral complete CHO on the left and a right-sided partial CHO due to intraoperative hemorrhage. Case 5 had a shortened ceratohyoid bone from a previous partial CHO procedure and underwent bilateral CHO.

Complications associated with the procedure included hemorrhage from branches of the lingual vessels in six (18%) horses. Three of these horses underwent bilateral CHO (50%) and three horses underwent unilateral CHO (50%). The bleeding resolved after the application of pressure (*n* = 3, 9%) or hemoclips (*n* = 1, 3%). Two cases experienced hemorrhage that did not resolve during surgery and resulted in emergency tracheostomy (both bilateral CHO). Other complications encountered included worsening (*n* = 1, 3%) or occurrence (*n* = 1, 3%) of facial nerve paralysis, which resolved 48 h postoperatively and did not resolve, respectively (both bilateral CHO).

Tracheostomy was performed in five (15%) horses (all bilateral CHO). Two (6%) were placed prior to surgery due to dysphagia and pharyngeal collapse, two (6%) were performed due to significant hemorrhage during surgery, and one (3%) was performed due to reduced airflow noted in recovery after extubation.

Due to significant ataxia, one (3%) horse (unilateral CHO) was induced to a sling. In addition to this horse, three (12%) more horses were recovered in a sling (one unilateral CHO and two bilateral CHO). Of these, one (3%) horse (unilateral CHO) was placed in a sling after a prolonged recovery, and the remaining two horses were placed in a sling for recovery prophylactically.

### 3.5. Short-Term Outcomes

The mean hospitalization duration was 7.5 days (range 2–28 days). Thirty-one of 33 (94%) horses survived to discharge. Of these, 17 of 17 (100%) horses underwent unilateral CHO, and 13 of 16 (81%) horses underwent bilateral CHO. Of the 31 horses that were discharged from the hospital, two (6%) cases were euthanized within 6 months for progressive ataxia or dysphagia (both bilateral CHO). Two (6%) cases died prior to 1 year for reasons unrelated to THO, and Case 33 had bilateral CHO performed less than 1 year (10.5 months) from the preparation of this manuscript.

### 3.6. Long-Term Outcomes

Twenty-six (84%) of 31 horses survived long-term (>1 year). A summary of outcomes is presented in Table 1.

### 3.7. Outcome of Horses with Bilateral Clinical Signs

All three horses that had bilateral clinical signs on presentation received bilateral CHO. Case 5 (endoscopy score grade 2, left; CT score grade 1, bilateral) was euthanized due to persistent ataxia 6 months after surgery. Case 6 (endoscopy score grade 3, bilateral; CT score grade 3, bilateral) greatly improved but its symptoms did not resolve due to persistent facial nerve paralysis and it retired prior to THO diagnosis. Case 33 (endoscopy score grade 1, left; CT score grade 1, bilateral) survived to discharge but retired due to persistent ataxia and could no longer be ridden.

### 3.8. Statistical Analysis Results

No significant differences were found between horses that underwent unilateral or bilateral CHO with respect to survival at 1 year, improvement in clinical signs, or return to work. There was no association between clinical signs and outcomes when unilateral and bilateral cases were analyzed separately using univariable logistic regression (Table 2).

A multivariable model could be constructed only when unilateral and bilateral CHO patients were combined based on clinical improvement alone (not on return to work) (Table 3). A univariable model for return to work identified age as the only predictor, with 17% lower odds of returning to work for each additional year of age (Table 4). For the multivariable model, head tilt, eyelid droop, sex, age, and duration of clinical signs were all included. After backward elimination, age and duration of clinical signs remain. Each additional year of age is associated with 28% lower odds of improvement, and each additional day of clinical signs before presentation is associated with 1.3% lower odds of improvement. These data should be treated as exploratory. There was no statistical difference in outcome between unilateral and bilateral CHO.

The severity of imaging changes was not associated with outcome. Analysis of endoscopic grades using a 0–3 scale decreased interobserver agreement, so statistical analysis was performed using grades 0–2, combining grade 1 (mild) and grade 2 (moderate).

## 4. Discussion

In this study, we report the short- and long-term outcomes of THO cases undergoing surgical treatment for unilateral versus bilateral CHO. To the authors’ knowledge, this is the first study to compare outcomes between unilateral and bilateral CHO. The current recommendation is to perform unilateral CHO on the most affected side and proceed only with a second surgery when clinical signs persist or recur on the opposite side. Only a few cases of bilateral CHO during a single anesthetic event have been described in the literature [21]. Still, there are multiple reports of horses undergoing two unilateral CHO procedures due to persistent signs or occurrence of signs on the contralateral side [21,23]. Historically, performing a partial stylohyoidectomy bilaterally was reported to result in difficulty with prehension following surgery [12]. Because of this, concern for causing dysphagia or difficulty with mastication has been raised after performing bilateral CHO, but this complication has not been reported. In all cases in this study undergoing bilateral CHO, there were no signs of difficulty with prehension, dysphagia, or destabilization of the hyoid apparatus following surgery. In our study, cases with the bilateral signs of THO on endoscopy and/or CT evaluation strongly contributed to the decision to perform a bilateral CHO. In addition, Case 5 continued to show clinical signs after a previous partial unilateral CHO but improved greatly after a second surgery to remove both ceratohyoid bones fully.

Although no specific complications related to a bilateral approach were documented, we advised the owners to restrict exercise for 3 to 4 months following surgery to allow the surgical site to fully heal and form a fibrous scar. Although not clinically reported, retroversion of the epiglottis during strenuous exercise after simultaneous removal of both ceratohyoid bones could be a potential complication. The stability of the epiglottis during exercise is maintained by the hyoid apparatus and tongue movement, as well as inspiratory contraction of the hyoepiglotticus and geniohyoid muscles, which are innervated by the hypoglossal nerve. Holcomb et al. 1997 demonstrated that epiglottic retroversion can be induced by anesthetizing the hypoglossal nerves within the guttural pouches [26]. In addition, Cheetham et al. 2009 evaluated the effect of bilateral hypoglossal nerve block at the level of the ceratohyoid bone [27]. When horses were exercised on a treadmill post block, two out of ten horses showed retroversion of the epiglottis [27]. During bilateral CHO, irritation of both hypoglossal nerves or the paired geniohyoid muscles may occur, potentially destabilizing the epiglottis during exercise in the early postoperative phase. In addition, epiglottis instability could be introduced in the early stages after surgery due to bilateral disruption of the bony connection between the basihyoid and stylohyoid bones.

Median imaging grades for both modalities were determined by averaging the observer grades rather than by consensus to avoid bias in scoring. Combining endoscopic scores increased interobserver agreement. Retrospective grading had no impact on the outcome, and the current grading systems allow for significant subjectivity. It would be valuable to develop a more formal endoscopic grading system to better understand the impact of imaging findings on overall prognosis. In addition, changes to the THJ are not always visible endoscopically and may require advanced imaging. In this study, retrospective CT image grading revealed bilateral THJ changes in all but one case. This contrasts with the preoperative CT reports (62% documented bilateral findings), on which the decision to perform unilateral versus bilateral surgery was based. The grading system of Tanner et al. was published in 2018 and was not available for earlier cases of this study. It is now widely used by our radiologists and influences our decision-making on unilateral versus bilateral surgery. Further, the authors have become more comfortable advocating for a bilateral CHO, even though horses often present with only unilateral clinical signs.

This study reported a long-term survival rate of 84%. Eighty-six percent of those cases showed improvement, with 38% showing complete resolution of signs. Seventy-seven percent of horses were able to return to work, with 62% returning to their previous level of exercise. This survival rate was comparable to another study that reported 89% survival but also reported a lower return-to-work rate of 50% [28]. Our return-to-work rate was comparable to other studies, with one showing a 65% return to use [21] and another showing an 80% return to exercise [22]. These studies evaluated smaller sample sizes or had varied durations of clinical signs or patient ages compared to the cohort reported here.

Based on statistical analysis, the duration of clinical signs and age were the only predictors of outcome: with each additional year of age, horses were less likely to return to work, and a longer duration of signs was associated with a lower likelihood of improvement. It is important to note that some of the horses that did not return to work were either already retired or close to retirement age, but when signs are recognized in younger horses, they are more likely to return to work. In this population, many horses, although not completely resolved, were able to return to some form of work or were pasture-sound, which aligned with many owners’ expectations regarding quality of life. THO is a progressive, degenerative disease process that can have longer-standing subclinical effects in older horses. This could contribute to incomplete resolution of ataxia or other peripheral nerve signs and reduce the horse’s ability to return to work. The long-term results may differ in younger horses, and this should be considered when discussing postoperative expectations with owners.

The complication rate was low in this study and did not differ significantly between the surgical groups. A previously reported complication in surgery that occurred in this study was intraoperative hemorrhage. Hemorrhage can be mild to severe and persist or recur during recovery, leading to blood accumulation in the soft tissues of the laryngeal region and airway obstruction [21,22]. Despite the lack of statistical significance, all cases receiving a tracheostomy underwent bilateral CHO, including two cases that required emergency tracheostomy following surgery due to uncontrolled hemorrhage. Case 18 developed significant hemorrhage from the left lingual artery branch while disarticulating the ceratohyoid–stylohyoid joint. The hemorrhage was controlled by compression, and the surgery site was closed. The horse recovered from anesthesia but, once standing, showed swelling of the throat latch and airway obstruction after extubation. Despite emergency tracheostomy, the airway could not be re-established, and the horse died as a consequence. Necropsy confirmed hemorrhage from the surgery site, leading to airway obstruction. Case 29 underwent bilateral CHO and experienced severe hemorrhage from the left lingual artery branch. The hemorrhage improved with direct pressure, and the surgical site was closed; however, the horse developed progressive swelling of the head and throat latch region during recovery. A tracheostomy was performed before extubation, and recovery from anesthesia was uneventful. The horse was treated with supportive care postoperatively, and the swelling resolved after 4 days. This horse returned to full athletic performance as a barrel racer. All efforts should be made to isolate the bleeding vessel and stop hemorrhage intraoperatively. These cases highlight the importance of considering tracheostomy if hemorrhage cannot be resolved or the risk of recurrence remains.

Due to the risk of airway obstruction during recovery, the authors routinely maintain orotracheal intubation after CHO surgery. This is even more important when performing bilateral surgery under the same anesthetic event due to possible tissue swelling near the larynx.

An additional consideration that may affect CHO outcome is related to surgical technique. All CHO surgeries evaluated in this study were performed at a single institution by a limited group of surgeons. Alternative surgical approaches to CHO have been described, including partial CHO [23] and disarticulation of the basi-ceratohyoid joint [29]. These approaches reduce the likelihood of iatrogenic injury to the hypoglossal nerve, the linguofacial vein, branches of the lingual nerve, and, importantly, the lingual artery. They can also be used when encountering abnormal morphology of the ceratohyoid bone, which can make complete removal difficult. These alternative techniques offer potentially shorter surgery times, making them more approachable to perform under standing sedation. In one study, basi-ceratohyoid disarticulation had good outcomes, with all cases showing improvement and 50% achieving full resolution of signs, and 83% returning to work [29]. Partial CHO risks the formation of pseudoankylosis of the basi-ceratohyoid joint, which can cause signs to recur or worsen [30]. In addition, performing these procedures under standing sedation may be difficult when horses are severely ataxic. Larger case studies across varying sample populations may be beneficial in guiding case selection for the different surgical techniques.

Limitations of the study include the retrospective nature and reliance on owners for follow-up information, who may underappreciate residual neurologic signs. Objective ataxia grading was not performed in every case before and after surgical treatment, making follow-up data more subjective. Reliance on medical records before the current system at this institution is also a limitation, as some older case histories may be incomplete. The small sample size of this study poses difficulties for drawing conclusions from the analysis and should be treated as exploratory. Also, surgical treatments were not randomized, and postoperative recommendations varied between treatment groups, which can skew the conclusions about outcomes. Given the exploratory nature of this study, long-term outcome was broadly characterized, and the overall positive outcome may also reflect different expectations for a mature population of horses. While this study uses a small sample size, this is the largest cohort examined to date for bilateral CHO, and the results, while offering insight into the outcomes of these cases, should be interpreted cautiously.

## 5. Conclusions

To the authors’ knowledge, this is the first study to directly compare horses undergoing unilateral or bilateral CHO, where almost half of the cases underwent bilateral CHO, with outcomes being similar between the two groups. This study emphasizes the advantages of CT imaging as an adjunct to surgical planning and that CT severity does not always correlate with outcome. In conclusion, the findings of this study support bilateral CHO as a feasible surgical option in carefully selected horses with bilateral imaging abnormalities, provided that appropriate postoperative exercise restriction is implemented.

## Figures and Tables

**Table 1:** Summary of each case including signalment (breed, weight [kg BW], age [years], and sex) as well as duration of clinical signs [days] prior to presentation, significant clinical signs on presentation, surgical procedure performed (unilateral or bilateral CHO), and overall outcome. * Case 33 had bilateral CHO performed less than 1 year from the preparation of the manuscript. “Unknown” indicates that this information was not available in the medical record.

Case	Breed	Weight (kg)	Age (Years)	Sex	Duration of Clinical Signs	Clinical Signs	Surgical Treatment (CHO)	Outcome
1	Welsh pony cross	375	15	mare	1	Head tilt, nystagmus, and ataxia	Unilateral	Ataxia resolved; intermittent head tilt persisted
2	Friesian	623	7	gelding	28	Purulent drainage from right ear	Unilateral	Complete resolution; returned to previous exercise
3	Thoroughbred	466	20	gelding	7	Facial n. paralysis, dysphagia, difficulty with bit, and corneal ulcer	Unilateral	Intermittent eyelid droop; returned to lower level of exercise
4	Paint	435	15	mare	12	Head tilt, facial n. paralysis, and ataxia	Bilateral	Improved significantly; safe enough to ride
5	Appaloosa	555	7	mare	28	Head tilt and loss of tongue tone	Bilateral	Euthanized d/t progressive signs of ataxia
6	Standardbred	562	13	gelding	1	Head tilt, masseter muscle atrophy, ataxia, and muzzle deviation	Bilateral	Significant improvement; mild muzzle deviation persisted; retired prior to surgery
7	Thoroughbred	525	13	gelding	365	Dysphagia	Unilateral	Complete resolution; returned to previous exercise
8	Quarter horse	533	12	mare	5	Facial n. paralysis	Unilateral	Complete resolution; returned to previous exercise
9	Thoroughbred	530	11	gelding	1	Head tilt and nystagmus	Unilateral	Complete resolution
10	Morgan	528	7	gelding	365	Head tilt and ataxia	Bilateral	Significant improvement; returned to previous exercise
11	Morgan	404	21	mare	30	Head tilt, muzzle deviation, and eyelid droop	Unilateral	Improvement without resolution; returned to previous exercise
12	Appaloosa	495	10	gelding	28	Facial n. paralysis, nystagmus, and corneal ulcer	Unilateral	Corneal ulcers resolved’ mild persistent facial n. paralysis; returned to previous exercise
13	Thoroughbred	446	12	mare	365	Head shaking and facial n. paralysis	Bilateral	Complete resolution; returned to previous exercise
14	Westphalian	620	14	gelding	1	Facial n. paralysis and ataxia	Unilateral	Complete resolution; returned to previous exercise
15	Warmblood	642	20	gelding	30	Facial n. paralysis	Unilateral	Mild ear droop; returned to previous exercise
16	Warmblood	639	20	mare	60	Abnormal head carriage	Unilateral	No improvement; returned to previous exercise
17	Draft cross	500	17	mare	150	Head tilt	Unilateral with contralateral disarticulation	Mild improvement; head tilt persisted; retired; euthanized unrelated to THO
18	Thoroughbred	557	21	gelding	11	Ataxia	Bilateral	Died in recovery
19	Thoroughbred	552	27	gelding	unknown	Facial n. paralysis with corneal ulcer	Bilateral	Returned to previous exercise
20	Mustang	414	5	mare	unknown	Poor performance	Unilateral	Complete resolution in 6 weeks; returned to previous exercise
21	Morgan	417	25	mare	7	Pharyngeal collapse	Bilateral	Improved; euthanized for colic 2 months later
22	Oldenburg	540	14	mare	1	Head tilt, nystagmus, and ataxia	Bilateral	Ataxia resolved; mild head tilt persisted; retired prior to surgery
23	Friesian cross	490	16	gelding	unknown	Head shaking and poor performance	Unilateral	Complete resolution; returned to previous exercise
24	Quarter horse	510	18	gelding	1	Difficulty chewing and ataxia	Unilateral	Complete resolution in 6 mo; returned to previous exercise
25	Thoroughbred	500	23	mare	3	Head tilt, nystagmus, and ataxia	Bilateral	Ataxia improved; retired
26	Quarter horse	600	15	mare	1	Dysphagia and pharyngeal collapse	Bilateral	Euthanized d/t presence of large mass in GP
27	Welsh pony	325	12	gelding	365	Abnormal head carriage, nystagmus, masseter m. atrophy, loss of tongue tone, dysphagia, and ataxia	Bilateral	Euthanized d/t persistent dysphagia
28	Donkey	119	24	jenny	10	Facial n. paralysis, masseter m. atrophy, and corneal ulcer	Bilateral	Facial n. paralysis improved
29	Quarter horse	524	12	gelding	60	Facial n. paralysis	Bilateral	Complete resolution; returned to barrel racing
30	Quarter horse	517	24	mare	30	Difficulty chewing and ataxia	Unilateral + condylectomy on ipsilateral side	Complete resolution; returned to previous exercise
31	Paint	390	17	mare	90	Ataxia	Bilateral	Complete resolution; returned to lower level exercise
32	Quarter horse	581	18	gelding	365	Poor performance and bit difficulties	Unilateral	Only ridden with bitless bridle; returned to lower level exercise
33	Arabian	431	28	gelding	56	Facial n. paralysis and ataxia	Bilateral	* Facial n. paralysis resolved; ataxia remained; retired

**Table 2:** Univariate logistic regression model describing the association between the explanatory variables and improvement of clinical signs following unilateral or bilateral CHO. Odds ratios and confidence intervals are for ‘yes’ outcomes, as ‘no’ was used as the referent outcome for all explanatory variables. *** Explanatory variables included in the multivariable model before backward model selection.

Explanatory Variable	Odds Ratio	95% Confidence Interval	*p* Value
Headshaking	1.038	0.070 to 150.942	0.982
Head tilt	0.267	0.030 to 1.894	0.183 ***
Abnormal head carriage	0.148	0.005 to 4.239	0.207
Ear drooping	1.895	0.235 to 39.938	0.591
Eyelid drooping	5.359	0.514 to 731.087	0.185 ***
Nystagmus	0.869	0.098 to 18.961	0.909
Muzzle deviation	2.222	0.279 to 46.676	0.501
Pharyngeal collapse	1.038	0.069 to 150.942	0.982
Masseter atrophy	1.510	0.118 to 214.163	0.786
Loss of tongue tone	0.308	0.023 to 7.504	0.378
Poor performance	1.510	0.118 to 214.163	0.786
Chewing difficulty	1.510	0.118 to 214.163	0.786
Dysphagia	2.020	0.171 to 282.572	0.628
Ataxia	0.370	0.043 to 2.591	0.318
Bit issues	1.078	0.073 to 156.920	0.963
Corneal ulcer	1.078	0.073 to 156.920	0.963
Bilateral signs *	0.840	0.035 to 133.809	0.921
Endoscopy score: Left	0.659	0.118 to 2.645	0.577
Endoscopy score: Right	1.207	0.338 to 4.902	0.769
Computed tomography score: Left	0.950	0.295 to 2.792	0.925
Computed tomography score: Right	1.755	0.488 to 6.670	0.376
Age	0.823	0.673–1.006	0.057 ***
Weight	1.004	0.994 to 1.013	0.396
Sex **	0.217	0.010 to 1.697	0.195 ***
Duration of clinical signs	0.994	0.986 to 1.000	0.071 ***

* Only horses with bilateral disease were included. ** Mares were used as the reference category.

**Table 3:** Univariate logistic regression model describing the association between the explanatory variables and return to work following unilateral or bilateral CHO. Odds ratios and confidence intervals are for ‘yes’ outcomes, as ‘no’ was used as the referent outcome for all explanatory variables.

Explanatory Variable	Odds Ratio	95% Confidence Interval	*p* Value
Headshaking	2.442	0.176 to 348.733	0.544
Head tilt	1.244	0.261 to 7.003	0.789
Abnormal head carriage	0.409	0.015 to 11.098	0.543
Ear drooping	1.021	0.209 to 5.807	0.981
Eyelid drooping	0.824	0.163 to 4.759	0.817
Nystagmus	2.5	0.333 to 51.710	0.433
Muzzle deviation	0.656	0.139 to 3.244	0.593
Pharyngeal collapse	0.409	0.015 to 11.099	0.543
Masseter atrophy	0.857	0.073 to 19.829	0.905
Loss of tongue tone	0.857	0.072 to 19.829	0.905
Poor performance	0.857	0.072 to 19.829	0.905
Chewing difficulty	1.510	0.118 to 214.163	0.786
Dysphagia	2.020	0.171 to 282.572	0.628
Ataxia	0.533	0.114 to 2.443	0.414
Bit issues	0.364	0.013 to 9.924	0.492
Corneal ulcer	0.350	0.053 to 2.270	0.258
Bilateral signs *	0.840	0.035 to 133.809	0.921
Endoscopy score: Left	1.426	0.527 to 4.195	0.487
Endoscopy score: Right	1.312	0.515 to 3.622	0.573
Computed tomography score: Left	0.899	0.384 to 2.008	0.796
Computed tomography score: Right	1.458	0.557 to 3.937	0.437
Age	0.831	0.692 to 0.961	0.023
Weight	0.999	0.991 to 1.001	0.950
Sex **	1.950	0.438 to 9.488	0.386
Duration of clinical signs	1.000	0.994 to 1.007	0.983

* Only horses with bilateral disease were included. ** Mares were used as the referent category.

**Table 4:** Multivariable logistic regression model describing the association between the explanatory variables and clinical improvement following unilateral or bilateral CHO. Odds ratios and confidence intervals are for ‘yes’ outcomes, as ‘no’ was used as the referent outcome for all explanatory variables.

Explanatory Variable	Odds Ratio	95% Confidence Interval	*p* Value
Age	0.717	0.410 to 0.957	0.019
Duration of clinical signs	0.987	0.968 to 0.997	0.007

## Data Availability

The raw data supporting the conclusions of this article will be made available by the authors on request.

## Abbreviations

CT, computed tomography; NSAID, non-steroidal anti-inflammatory; THJ, temporohyoid joint; THO, temporohyoid osteoarthropathy; CHO, ceratohyoidectomy.
